# Metabolic Syndrome-Driven Changes in Cardiac Lymphatic Endothelium: mRNA Expression and Emerging Questions

**DOI:** 10.3390/pathophysiology33010004

**Published:** 2026-01-09

**Authors:** Ewa Jankowska-Steifer, Anna Ratajska, Aleksandra Flaht-Zabost, Dorota Magdalena Radomska-Leśniewska, Iwona Badurek, Ewelina Kiernozek, Aneta Moskalik, Barbara Majchrzak, Mateusz Bartkowiak, Krzysztof Bartkowiak, Bogdan Ciszek, Marek Kujawa, Justyna Niderla-Bielinska

**Affiliations:** 1 Department of Histology and Embryology, Collegium Anatomicum, Medical University of Warsaw, Chałubińskiego 5 Str, 02-004 Warsaw, Poland; ewa.jankowska-steifer@wum.edu.pl (E.J.-S.);; 2 Department of Pathology, Medical University of Warsaw, Collegium Anatomicum, Chałubińskiego 5 Str, 02-004 Warsaw, Poland; anna.ratajska@wum.edu.pl (A.R.);; 3 Department of Immunology, University of Warsaw, Miecznikowa 1 Str, 02-096 Warsaw, Poland; 4 Postgraduate School of Molecular Medicine, Medical University of Warsaw, 02-097 Warsaw, Poland; aneta.moskalik@gmail.com; 5 Department of General, Transplantation and Liver Surgery, Medical University of Warsaw, Banacha 1a Str, 02-097 Warsaw, Poland; 6 Department of Clinical Anatomy, Medical University of Warsaw, Collegium Anatomicum, Chałubińskiego 5 Str, 02-004 Warsaw, Poland; 7 Department of Histology and Embryology, Faculty of Medicine, Lazarski University, 02-662 Warsaw, Poland

**Keywords:** metabolic syndrome, lymphatic endothelial cells, mRNA, cardiac lymphatic vessels, lymphangiogenesis, apoptosis, fibrosis, cell metabolism

## Abstract

**Background/Objectives**: Metabolic syndrome (MetS) conditions lead to structural and functional alterations in cardiomyocytes, microvasculature, and extracellular matrix (ECM), leading to myocardial fibrosis and impaired diastolic function. Cardiac lymphatic vessels (LVs) are increasingly recognized as key regulators of myocardial homeostasis, yet their response to MetS remains poorly understood. Therefore, we aimed to investigate transcriptional changes in cardiac lymphatic endothelial cells (LECs) in db/db mice, a well-established model of MetS. **Methods**: Using flow cytometry-sorted LECs and RT-PCR, we analyzed mRNA expression of genes involved in lymphangiogenesis, metabolism, mechanotransduction, immune cell trafficking, and ECM interactions. **Results**: Our findings show the transcriptional plasticity of cardiac LECs in response to MetS. **Conclusions**: Although our study is limited by the lack of protein-level validation and functional assays, our approach provides a broader interpretative framework and identifies potential directions for future research, including functional studies and pathway-specific investigations of the identified genes to assess their impact on lymphatic flow and cardiac function. Understanding LEC responses to metabolic stress may uncover novel therapeutic targets for heart failure associated with MetS.

## 1. Introduction

The cardiac lymphatic system plays a crucial role in maintaining myocardial homeostasis by draining interstitial fluid and regulating immune cell trafficking during inflammation [1,2]. Despite increasing recognition of its importance, the impact of metabolic syndrome (MetS) and its associated comorbidities on cardiac lymphatic vessels (LVs) remains poorly understood. MetS is defined by a cluster of risk factors, including obesity, insulin resistance/type 2 diabetes mellitus, dyslipidemia, hypertension, and chronic low-grade inflammation [3]. Each of these components contributes to the development of heart failure (HF) and worsens clinical outcomes.

The pathogenesis of HF in MetS involves structural and functional alterations in cardiomyocytes, microvasculature, and extracellular matrix (ECM), leading to myocardial fibrosis and impaired diastolic function [4,5]. Not only the quantity but also the quality of fibrotic tissue—including increased collagen cross-linking and a shift toward rigid collagen I—contributes to myocardial stiffness and dysfunction. Recent studies suggest that lymphatic dysfunction may be a key driver of this process, as impaired lymphatic drainage promotes interstitial edema and fibroblast activation [6,7].

Db/db mice, exhibiting leptin receptor mutation, present obesity, type 2 diabetes mellitus, high blood glucose, dyslipidemia, and nondipping circadian blood pressure pattern; therefore, they are a representative animal model of MetS [8,9,10]. In this strain, structural remodeling of cardiac LVs has been documented, including reduced vessel density, altered endothelial morphology, widened junctional gaps, decreased vesicular transport, and thickened basement membranes—all indicative of a leaky and dysfunctional lymphatic phenotype [11]. Furthermore, the microvascular paradigm proposes that systemic inflammation and endothelial dysfunction underlie the development of HF with preserved ejection fraction (HFpEF) in MetS [12,13]. These findings support the hypothesis that cardiac lymphatics actively respond to metabolic and inflammatory stress and may contribute to myocardial remodeling in MetS.

While most studies focus on blood vascular endothelial cells (BECs), lymphatic endothelial cells (LECs) may be similarly affected by the metabolic environment, leading to impaired lymph flow and altered vessel integrity [14,15]. Therefore, the aim of this study was to investigate whether MetS induces transcriptional changes within cardiac LECs that may accompany structural remodeling, previously described by our group [11]. To address this, we analyzed the mRNA expression levels of selected genes in myocardial LECs isolated from db/db mice. The selected transcripts were associated with key biological processes relevant to lymphatic vessel function, including lymphangiogenesis, endothelial metabolism, barrier integrity, ECM interactions, immune cell trafficking, and lymph flow regulation. Additionally, we examined the localization of selected proteins within cardiac LVs to better understand the morphological consequences of transcriptional switch. Although the transcriptomic approach was exploratory, the selection of genes was biologically guided by published evidence and by our previous observations of lymphatic remodeling in the db/db heart. The chosen genes represent key regulatory nodes within pathways most likely to be influenced by the metabolic environment characteristic of MetS. Understanding the molecular and structural changes in cardiac LECs under MetS conditions may provide new insights into the mechanisms of HF and identify novel therapeutic targets.

## 2. Materials and Methods

### 2.1. Animals

This study was performed on BKS.Cg-Dock7<m>+/+Lepr<db>/J male mice (db/db); the C57BL/6J strain was used as control (16 db/db mice and 16 controls). All animal experiments were approved by the First Local Bioethics Committee of the University of Warsaw, Poland, and carried out in accordance with EU Directive 2010/63/EU for animal experiments. Nine-week-old male mice were purchased from Charles River (Sant’Angelo Lodigiano, Italy) and kept under specific pathogen-free conditions, with unlimited access to LabDiet^®^ 5K52 (6% fat) chow (Charles River Laboratory, Sant’Angelo Lodigiano, Italy). At the age of 21 weeks, the animals were sacrificed by CO_2_ asphyxiation, and their hearts were isolated for further analysis. A total of 28 hearts were collected for FACS analysis—14 from db/db mice and 14 from control mice. Additionally, 4 hearts (2 from db/db and 2 from control mice) were snap-frozen for immunostaining.

### 2.2. Lymphatic Endothelial Cell Isolation by Flow Cytometry Sorting

**Figure 1:**
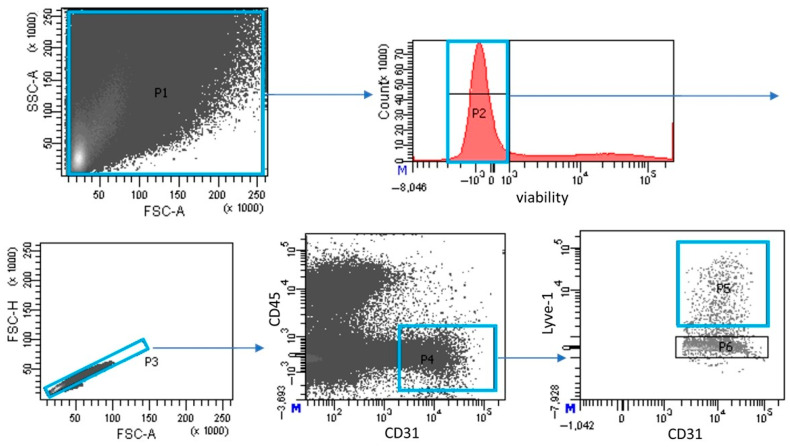
Sorting strategy of cardiac LECs from single-cell suspension. Cells obtained from the cardiac tissue (gate P1). Dead cells were excluded (gate P2). Next, doublets were excluded (gate P3). Within living and single cells, a CD31-positive and a CD45-negative population was selected (gate P4). Finally, Lyve-1+/CD31+/CD45− cells were sorted (gate P5).

Hearts were cut in half and rinsed in PBS to wash out blood from heart chambers. Next, the hearts were cut into small pieces and digested with 0.5 mg/mL collagenase type II (Sigma-Aldrich, St. Louis, MO, USA) on a magnetic stirrer at 37 °C for 45 min. To obtain single-cell suspensions, the digested tissue was pipetted and filtered through a 40 μM nylon filter (Falcon, Corning, New York, NY, USA). The pooled amount of LEC from two hearts was used for one separate FACS sorting. The cells were washed twice and suspended in a staining buffer (1% BSA in PBS). First, the cells were incubated with Fixable Viability Dye (eBioscience, San Diego, CA, USA, cat. No. 65-0865-14, Thermo Fisher Scientific, Waltham, MA, USA). The antibodies were as listed: Lyve-1 (clone: 223322, cat. no. FAB2125P, R&D Systems, Minneapolis, MN, USA), CD31 (clone 390, cat. no. 563356, BD Biosciences, San Jose, CA, USA), and CD45 (clone 30-F11, cat. no. 563891, BD Biosciences, San Jose, CA, USA). Stained cells were washed, suspended in PBS, sorted with FACS Aria I and analyzed with BD FACSDiva software v7.0 (Becton Dickinson, Franklin Lakes, NJ, USA). LEC were identified as LYVE-1+/CD31+/CD45−. The sorting strategy is shown in Figure 1.

### 2.3. RT-PCR Analysis

RNA was isolated with an RNeasy Micro Kit (Qiagen, Venlo, The Netherlands, cat. no. 74004) according to the manufacturer’s protocol. Concentration and quality of RNA were assessed with a NanoDrop spectrophotometer. The RNA was transcribed into cDNA according to the manufacturer’s protocol (High-Capacity cDNA Reverse Transcription Kit, Thermo Fisher Scientific). Preamplification was performed with TaqMan™ PreAmp Master Mix Kit (ThermoFisher Scientific, cat. no. 4384267). Relative quantification of gene expression was performed using Real-time PCR at Abi Prism 7500 (Thermo Fisher Scientific) in 96-well optical plates. Gapdh was used as an endogenous control (Mm99999915_g1). Each sample was run in triplicate. To detect mRNA levels of selected genes, the following TaqMan Gene Expression Assays were used: CD36: Mm00432403_m1; Pten: Mm00477208_m1; Foxo1: Mm00490671_m1; Klf2: Mm00500486_g1; Bax: Mm00432051_m1; Bcl2: Mm00477631_m1; Cpt1a: Mm01231183_m1; Ccl21a: Mm03646971_gH; Cx3cl1: Mm00436454_m1; Icam1: Mm00516023_m1; Vcam1: Mm01320970_m1; Emilin1: Mm00467244_m1; Mmp2: Mm00439498_m1; Reln: Mm00465200_m1; Snai1: Mm00441533_g1; Snai2: Mm00441531_m1; Acta2: Mm00725412_s1; Cdh5: Mm00546194_s1; Gata2: Mm00492301_m1; Cldn5: Mm00727012_s1; Ceacam1: Mm04204476_m1; Pdpn: Mm01348912_g1; Fbn1: Mm00435217_m1; Nos3: Mm00435217_m1; Sdc4: Mm00488527_m1. The reactions were performed in a 20 μL volume with TaqMan Universal PCR Master Mix (Thermo Fisher Scientific), a primer set, an MGB probe and 5 ng of cDNA template. The thermal conditions were 10 min at 95 °C and 40 cycles of 15 s at 95 °C and 1 min at 60 °C. Sequence detection software v1.2 (Thermo Fisher Scientific) was used for analysis. Gene expression was measured with the relative quantification (RQ) with a comparative CT assay [16].

For RT-PCR data, the Mann–Whitney U test was applied with GraphPad Prism version 8.4.3 software; graphs were also prepared with GraphPad Prism version 8.4.3 software. Differences were considered statistically significant with *p*-value < 0.05.

### 2.4. Immunofluorescence Staining and Confocal Microscopic Analysis

A series of 10 µm frozen heart sections was fixed in 4% paraformaldehyde; washed with PBS; incubated with 1% BSA, 0.1% TritonX-100, and 0.1 M glycine in PBS for 30 min; and blocked with 10% donkey serum (Jackson ImmunoResearch Laboratories, West Grove, PA, USA). Sections were stained with a cocktail of various combinations of antibodies to different antigens: Lyve-1 (R&D Systems, cat. no. MAB2125; Angiobio, San Diego, CA, USA, cat. no. 11-034, final concentration 1:300), Podoplanin (R&D Systems, cat. no. AF3244, final concentration 1:50), CD31 (BD Biosciences, cat. no. 550274, final concentration 1:100), CD31 (R&D Systems, cat. no. AF3628, final concentration 1:100), Collagen III (Novusbio, Centennial, CO, USA, cat. no. NBP1-26547, final concentration 1:10), Collagen I (Abcam, Cambridge, UK, cat. no. ab34710, final concentration 1:150), SMA (Abcam, cat. no. ab21027, final concentration 1:100), syndecan 4 (Invitrogen, Waltham, MA, USA, cat. no. PA1-32485, final concentration 1:40) diluted in PBS with 5% donkey serum for 1 h at room temperature or for 12 h in 4 °C. After washing with PBS (2 × 10 min), the sections were incubated with donkey secondary antibodies (Cy™3-conjugated donkey anti-rabbit IgG, Jackson Immunoresearch, cat #711-165-152, final concentration 1:800; AlexaFluor™647 donkey anti-rat, Jackson Immunoresearch, cat #712-605-153, final concentration 1:500; FITC-conjugated donkey anti-goat, Jackson Immunoresearch, cat #705-095-147, final concentration 1:200) for 1 h. Cell nuclei were counterstained with DAPI (Thermo Fisher Scientific, Waltham, MA, USA). Sections mounted in Fluorescence Mounting Medium (Dako, Glostrup, Denmark) were viewed under a Leica confocal microscope (Leica, Wetzlar, Germany) and/or an Olympus confocal microscope (Olympus, Hachioji, Tokyo, Japan). Imaging was performed using Leica HC PL APO ×20/0.7 (Leica, Wetzlar, Germany) and Olympus SPlan APO ×20/0.7 (Olympus) objectives. Due to the use of variable camera zoom settings during image acquisition, exact magnification values cannot be provided; however, scale bars are included in all images.

### 2.5. The Use of Graphics Preparation

Graphical abstract was created by the authors with the use of image elements provided by Servier Medical Art (https://smart.servier.com/), licensed under CC BY 4.0 (https://creativecommons.org/licenses/by/4.0/) accessed on 28 October 2025.

## 3. Results

### 3.1. mRNA Analysis Shows Alteration in mRNA Expression for Selected Genes in LVs Endothelial Cells

**Figure 2:**
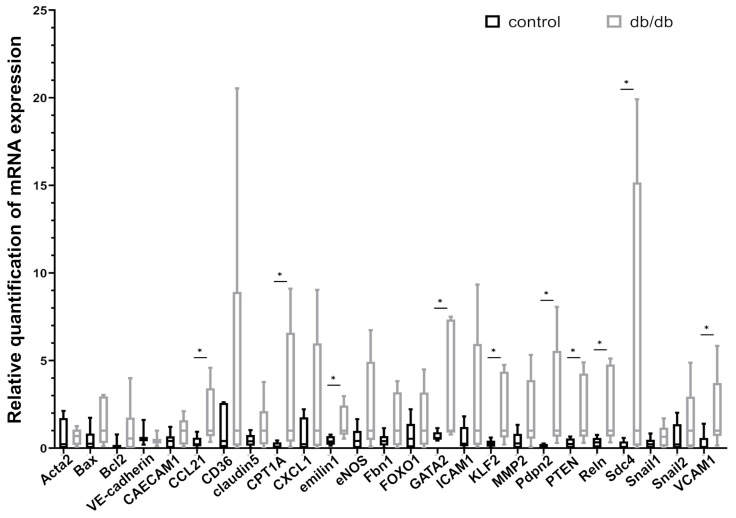
RT-PCR analysis. Selected mRNA expression in sorted LECs from db/db and control mice hearts. Some mRNA levels significantly increase in db/db mice compared to controls. Statistically significant differences (*p*-value < 0.05) are marked (*). Abbreviations: Acta2—Actin 2/smooth muscle actin; Bax—BCL2 Associated X; Bcl2—B-cell CLL/lymphoma 2; CCL21—Chemokine (C-C motif) ligand 21; CEACAM1—Carcinoembryonic antigen-related cell adhesion molecule 1; CPT1A—Carnitine Palmitoyltransferase 1A; CX3CL1—chemokine (C-X3-C motif) ligand 1; eNOS—endothelial nitric oxide synthase;Fbn1—fibrillin-1; FOXO1—Forkhead Box O1; GATA2—GATA-binding factor 2; ICAM-1—intercellular adhesion molecule 1; KLF2—Krüppel-like Factor 2; MMP2—Metalloproteinase 2; PDPN—podoplanin; PTEN—phosphatase and tensin homolog deleted on chromosome ten; Reln—reelin; Sdc4—syndecan 4; Snail1—Snail family transcriptional repressor-1; Snail2—Snail family transcriptional repressor-2; VCAM-1—vascular cell adhesion molecule 1; VE-cadherin—Vascular Endothelial Cadherin.

We used RT-PCR to measure the expression of selected mRNAs whose products are responsible for regulation of key activities/functions and phenotypic character of LVs, such as lymphangiogenesis, apoptosis, metabolism, inflammatory response, intercellular junctions (maintaining integrity of LV wall), lymphatic valve remodeling, molecules anchoring LEC to ECM, and endothelial-to-mesenchymal transition. Results of RT-PCR showed increased levels of mRNA expression of the following genes: KLF2, CPT1a, podoplanin (Pdpn), PTEN, VCAM1, CCL21, GATA2, emilin1, reelin (Reln) and Syndecan 4 (Sdc4) in db/db mice compared to control animals, whereas there were no marked differences in the levels of mRNA expression of CD36, FOXO1, eNOS, Bax, Bcl2, ICAM, CX3CL1, claudin 5, VE-cadherin, Ceacam1, fibrillin-1 (Fbn1), MMP2, Snail 1, Snail 2, and Acta2 (actin alpha 2 = alpha-smooth muscle actin or α-SMA) (Figure 2, Table 1).

### 3.2. Immunoconfocal Analysis of Lymphatic Vessel Shapes and Phenotypic Alterations

**Figure 3:**
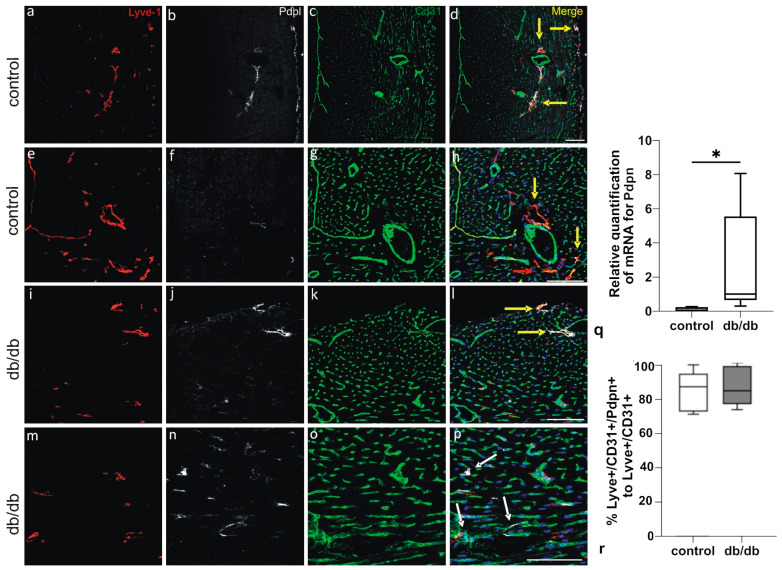
LVs localized subepicardially and perivascularly in left ventricles of hearts from control (**a**–**h**) and db/db (**i**–**p**) mice. Most of the lymphatic vessels express all three markers: Lyve-1 (red), Pdpn (white) and CD31 (green) (merge; yellow arrows); few Pdpn-positive cells do not express Lyve-1 or CD31 (merge; white arrows). Merged panels (**d**,**h**,**l**,**p**) include DAPI staining of nuclei (blue). (**m**–**p**) shows zoomed region of (**i**–**l**). (**q**) shows relative quantification of mRNA level for Pdpn. (**r**) presents the percentage of Pdpn-positive LVs among all cardiac LVs, as shown in representative confocal microscope images. Scale bars—100 µm. Abbreviations: LV—lymphatic vessel, Pdpn—podoplanin. Statistically significant differences (*p*-value < 0.05) are marked (*).

**Figure 4:**
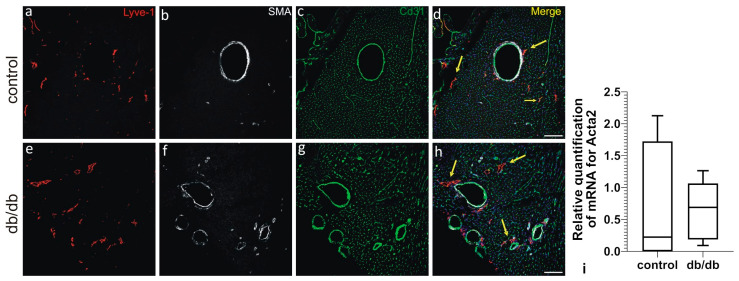
LVs located adjacent to coronary arteries in hearts from control (**a**–**d**) and db/db (**e**–**h**) mice. Lyve-1+/CD31+ lymphatic vessels (arrows) do not express SMA. Merged panels (**d**,**h**) include DAPI staining of nuclei (blue). (**i**) shows relative quantification of mRNA level for Acta2. Scale bars—50 µm. Abbreviations: Acta2—Actin 2/smooth muscle actin; SMA—smooth muscle actin.

**Figure 5:**
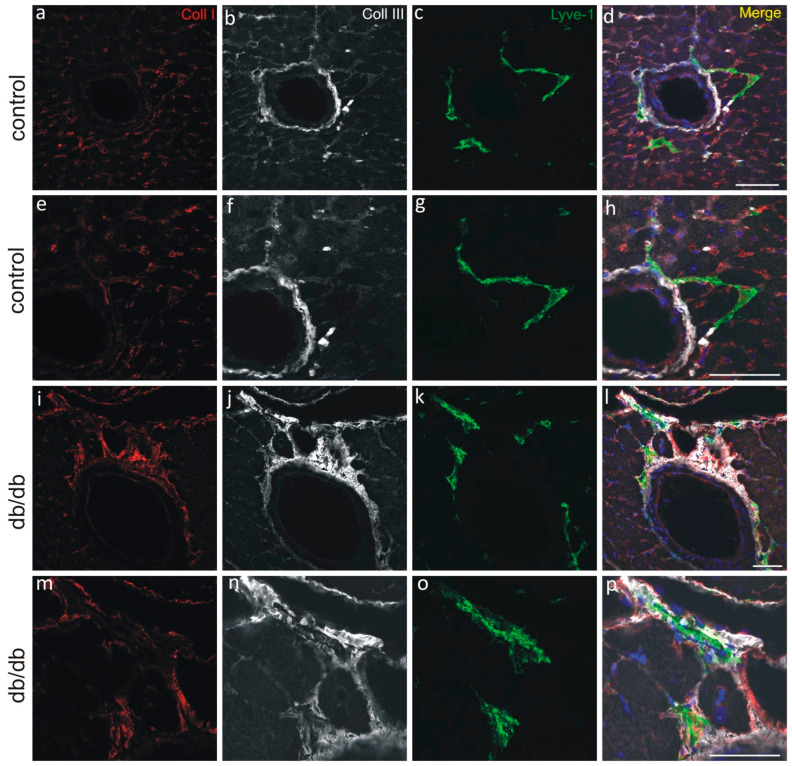
Myocardial perivascular LVs surrounded by collagen I and collagen III in control (**a**–**h**) and db/db (**i**–**p**) mice. Merged panels (**d**,**h**,**l**,**p**) include DAPI staining of nuclei (blue). (**e**–**h**) shows zoomed region of (**a**–**d**). (**m**–**p**) shows zoomed region of (**i**–**l**). Scale bar—50 µm.

**Figure 6:**
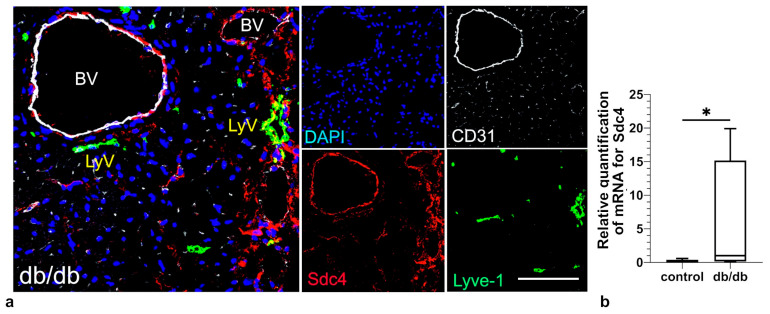
Myocardial perivascular LVs express Sdc4 (red) in db/db mice (**a**). (**b**) shows relative quantification of mRNA level for Sdc4. Scale bar—50 µm. Abbreviations: Sdc4—syndecan 4. Statistically significant differences (*p*-value < 0.05) are marked (*).

Multiple labeling of cardiac cryosections with lymphatic markers—Lymphatic vessel endothelial hyaluronan receptor 1 (LYVE-1), CD31, and PDPN—reveals that cardiac LVs are located subepicardially, myocardially (i.e., in the subepicardial half of the myocardial wall), and perivascularly in both control and db/db mice. Of note, not all LYVE-1+/CD31+ LVs express PDPN. In addition to the LYVE-1+/PDPN+/CD31+ and LYVE-1+/PDPN-/CD31+ vessels, we also observe PDPN-positive cells that do not express LYVE-1 or CD31 (Figure 3). CD31 was included as a pan-endothelial marker to identify all vascular structures, while LYVE-1 served as a specific marker for distinguishing lymphatic vessels from blood vessels. Immunostaining with anti-alpha-SMA reveals that LVs of db/db mice do not express this protein (Figure 4). Alpha-SMA was analyzed to assess the potential occurrence of endothelial-to-mesenchymal transition (EndoMT), as its upregulation in endothelial cells is considered a hallmark of this process. Collagen I and III are abundantly present around lymphatic vessels in db/db mice, similarly to Sdc4 (Figure 5 and Figure 6).

## 4. Discussion

Cardiac lymphatic vessels are increasingly recognized as dynamic structures that respond to metabolic and inflammatory cues. In the context of MetS, their dysfunction may contribute to myocardial remodeling through impaired fluid drainage, altered immune cell trafficking, and changes in ECM interactions. Recent studies have highlighted that LECs possess a unique metabolic and transcriptional profile, which is dynamically regulated during lymphangiogenesis and in response to pathological stimuli [15].

In this study, we investigated whether MetS-related conditions—obesity, hyperglycemia, and dyslipidemia—alter the transcriptional phenotype of myocardial LECs. Our aim was to identify molecular features that may reflect lymphatic remodeling in response to the metabolic environment. Because this is an exploratory transcriptomic analysis, the Discussion Section integrates our findings with current knowledge of lymphatic biology to provide plausible mechanistic interpretations. This broader scope is necessary, as many of the pathways suggested by the transcriptomic shifts cannot be fully resolved without complementary protein-level or functional studies.

It is important to note that our analysis is based on mRNA expression and does not include protein validation or functional assays. Given that mRNA abundance does not always reflect protein levels—due to factors such as post-transcriptional regulation, variable translation efficiency, and protein degradation—future work will be required to confirm the relevance of specific targets at the protein level and to determine their functional impact. The transcripts discussed were selected for their relevance to lymphatic vessel biology and their alterations in the MetS model. For this reason, the Discussion Section refers to pathways and mechanisms not directly measured in this study in order to situate our findings within a coherent biological framework. Finally, the breadth and length of the Discussion Section are intentional. Cardiac lymphatic endothelial biology in the context of metabolic disease remains a relatively underexplored field, and the available mechanistic literature is limited. As a result, a broader interpretative approach is necessary to contextualize the transcriptomic data, avoid over-interpretation, and outline biologically meaningful hypotheses. The extended Discussion Section therefore serves to connect our exploratory findings with established concepts in lymphatic research and to identify informed directions for future, hypothesis-driven studies.

### 4.1. Mechanosensation and Mechanotransduction of Lymphatic Vessels and Matrix Stiffness

Mechanotransduction, the conversion of mechanical stimuli such as fluid shear stress (FSS) and matrix stiffness into biochemical signals, regulates LEC gene expression, cytoskeletal dynamics, and junctional integrity [17,18]. In our model, we detected increased expression of mRNA for GATA-binding factor 2 (GATA2), Krüppel-like Factor 2 (KLF2), phosphatase and tensin homolog deleted on chromosome ten (PTEN) and PDPN, all of which are associated with mechanosensitive pathways. GATA2 is essential for lymphatic valve and vessel patterning, and its mutations cause primary lymphedema [19]. Importantly, GATA2 regulates the expression of junctional proteins via miR-126, and loss of GATA2 or miR-126 leads to defects in vascular endothelial cadherin (VE-cadherin) and claudin-5 expression. However, in our model, VE-cadherin and claudin-5 levels remained unchanged, suggesting that junctional integrity is preserved despite transcriptional activation of GATA2.

Although VE-cadherin expression remained unchanged in db/db mice compared to controls, its functional role in LEC remains critical. VE-cadherin acts as a mechanosensory adaptor and scaffolding protein that stabilizes vascular endothelial growth factor receptor 3 (VEGFR3) at the plasma membrane, enabling its activation by vascular endothelial growth factor C (VEGF-C) and adrenomedullin [20]. This interaction establishes a pro-lymphangiogenic signaling node essential for cardiac lymphatic maintenance. Through this mechanism, VE-cadherin facilitates downstream activation of phosphatidylinositol 3-kinase (PI3K) and other signaling pathways that regulate LEC proliferation, migration, and vessel integrity [21]. In this context, elevated PTEN, a lipid phosphatase that antagonizes PI3K signaling, may act as an intracellular brake, limiting VEGFR3-mediated lymphangiogenesis. Conditional deletion of PTEN in LECs has been shown to enhance lymphatic expansion and function without inducing inflammation, highlighting its role in fine-tuning VEGF-C responses [22].

Our study shows that not only is mRNA for PDPN elevated in db/db mice, but PDPN-positive LECs are also present within cardiac sections. Moreover, we observed a number of PDPN-positive cells that do not express LV markers. PDPN is a mucin-type transmembrane glycoprotein physiologically expressed by cardiac lymphatic endothelial cells, where it plays a critical role in lymphatic development and homeostasis. Following myocardial infarction (MI), PDPN expression expands beyond its typical localization, appearing in a heterogeneous population of progenitor cells. These cells may acquire PDPN in response to inflammatory stimuli, suggesting a dynamic role in post-ischemic remodeling. Notably, perivascular platelet-derived growth factor receptor beta (PDGFRβ)-positive cells in the chronic phase of MI co-express PDPN and prospero homeobox 1 (PROX-1), indicating potential transdifferentiation into lymphatic endothelial cells [23]. Our results may indicate that PDPN expression is not only connected with myocardium remodeling but also with progenitor activation.

Fbn-1, another key structural component of extracellular microfibrils and anchoring filaments in LVs, is produced by LEC and plays a role in maintaining vessel integrity and mechanotransduction [24,25]. In our model, expression of mRNA for Fbn1-1 remained unchanged, suggesting that the structural scaffold of the lymphatic ECM is preserved.

In addition, emillin1 mRNA levels were elevated in db/db mice. Emillin1 is an ECM glycoprotein involved in elastic fiber assembly and modulation of matrix tension [26]. Its upregulation may reflect an adaptive response to increased collagen deposition and ECM stiffness, which are known features of the myocardial environment in MetS. This suggests that emilin-1 may contribute to mechanotransduction signaling in LECs under conditions of altered matrix composition. Our study shows that collagen depositions are present around LVs (Figure 5), which further supports that mRNA for emilin 1 expression may be an answer to rising tension.

In the context of increased GATA2 and KLF2 expression, the preserved levels of VE-cadherin may allow continued VEGFR3-mediated signaling despite structural lymphatic defects. However, unchanged expression of mRNA for claudin-5 and metalloproteinase 2 (MMP2) suggests that junctional and matrix-remodeling responses are insufficient to compensate for impaired lymphatic architecture in the stiffened myocardial environment of db/db mice.

In our study, we also observed the increase in mRNA for Sdc-4 expression (Figure 6). Sdc-4, a transmembrane proteoglycan, is a well-established mechanosensor that regulates cellular responses to mechanical stimuli at the cell–matrix interface, including focal adhesion formation, integrin signaling, and subcellular localization of mechanistic target of rapamycin complex 2 (mTORC2) and Akt activation in vascular endothelial cells [27]. Sdc-4 has been shown to regulate cell polarity and valve morphogenesis via VANGL planar cell polarity protein 2 (VANGL2), suggesting a role in flow-dependent structural organization [28]. Although its mechanosensory function has been primarily described in blood vessels, it is plausible that Sdc-4 contributes similarly to mechanotransduction in LV, potentially influencing their integrity and remodeling under conditions of altered flow or increased ECM stiffness.

Interestingly, MMP2 expression remained at levels comparable to controls, suggesting that matrix remodeling via gelatinase activity is not actively upregulated under these conditions. This observation contrasts with previous studies showing that MMP-2 and MMP-14 are upregulated in softer environments and facilitate LEC migration and tube formation [29]. The lack of MMP2 induction in our model may reflect the influence of increased ECM stiffness, which has been shown to modulate MMP expression and limit matrix-degrading activity. Thus, while the capacity for remodeling may be preserved, it does not appear to be enhanced in the stiffened myocardial environment of db/db mice.

Together, these findings suggest that in the stiffened myocardial environment of db/db mice, mechanotransduction pathways involving GATA2, KLF2, and PDPN are activated, potentially as a compensatory response to impaired lymphatic structure and flow. However, unchanged expression of junctional and matrix-remodeling genes indicates that these responses are insufficient to restore proper lymphatic remodeling and function.

### 4.2. Fatty Acid β-Oxidation as a Metabolic Driver of LEC

Fatty acid β-oxidation (FAO) has emerged as a critical metabolic pathway supporting the development and function of LECs, particularly under conditions of metabolic stress or limited glucose availability [30,31]. Although LECs primarily rely on glycolysis for ATP production, FAO plays a complementary and indispensable role in maintaining cellular homeostasis. Beyond energy generation, FAO contributes to the production of acetyl-CoA, a key metabolite involved in epigenetic regulation. Acetyl-CoA serves as a substrate for histone acetylation, mediated by the histone acetyltransferase P300, which facilitates transcription of lymphangiogenic genes such as VEGFR3 in cooperation with the transcription factor PROX1 [30,32].

The initiation of mitochondrial FAO depends on carnitine palmitoyltransferase 1A (CPT1A), which enables the transport of long-chain fatty acids into the mitochondrial matrix by converting acyl-CoA into acyl-carnitine [33]. In our study, we observed increased CPT1A mRNA expression in cardiac LECs from db/db mice, suggesting enhanced FAO activity. This metabolic shift may reflect an adaptive response to the lipid-rich and inflammatory environment characteristic of MetS.

Interestingly, CD36 expression remained unchanged, despite its known role in fatty acid uptake and junctional stability in LECs. Its deletion has been associated with lymphatic leakage, visceral adiposity, and impaired glucose disposal. Moreover, CD36 silencing reduces cellular respiration and destabilizes VE-cadherin junctions, indicating its role in maintaining both metabolic and structural homeostasis [34]. The lack of CD36 upregulation, alongside increased CPT1A, may suggest a compensatory mechanism favoring intracellular lipid utilization over transmembrane uptake. Importantly, CPT1A is transcriptionally regulated by PROX1, the master regulator of lymphatic endothelial identity [30]. PROX1 enhances CPT1A expression to increase acetyl-CoA production, which is used for histone acetylation at lymphangiogenic gene loci, including VEGFR3. This PROX1–CPT1A–acetyl-CoA axis links metabolic activity to epigenetic regulation of lymphatic fate and function [30,35].

Taken together, the increased CPT1A expression, unchanged CD36 levels, and reduced lymphatic vessel density may reflect a disrupted PROX1–VEGFR3 feedback loop, potentially driven by metabolic stress and mitochondrial dysfunction. This imbalance could compromise LEC fate maintenance and vessel integrity, contributing to the observed lymphatic rarefaction in the diabetic heart [11].

### 4.3. CPT1a and EndoMT in Cardiac Lymphatics

Endothelial-to-mesenchymal transition is a biological process in which endothelial cells lose their characteristic markers and functions, acquiring mesenchymal properties similar to fibroblasts. During EndoMT, cells downregulate endothelial markers such as VE-cadherin and claudin 5 and upregulate mesenchymal markers such as α-smooth muscle actin (α-SMA), Snail family transcriptional repressor-1 (Snail1), and Snail family transcriptional repressor-2 (Snail2) [36,37]. This transition increases cell motility, invasiveness, and the ability to produce ECM components. This process is triggered by factors such as transforming growth factor beta (TGF-β) and fibroblast growth factor 2 (FGF2) deficiency, chronic inflammation, and viral infection (e.g., KSHV in Kaposi’s sarcoma). The Notch and PDGFRβ signaling pathways, as well as Smad and non-Smad pathways, regulate EndoMT [38]. This phenomenon has been widely described in vascular endothelia [39]. It has also been observed in LECs and is increasingly recognized as a factor contributing to lymphatic vessel dysfunction in various pathological contexts [40]. During EndoMT, LECs lose their typical markers (such as LYVE1, PROX1, PDPN, VEGFR3) and acquire mesenchymal characteristics (e.g., expression of transgelin, vimentin, α-SMA), leading to increased motility, invasiveness, and altered vascular permeability [41]. Lymphatic EndoMT is associated with impaired drainage, tissue fibrosis, and impaired lymphatic vessel function in inflammatory conditions, lymphedema, certain cancers, and aging [42]. Both MetS and aging share key pathogenetic mechanisms, including chronic low-grade inflammation, endothelial dysfunction, and impaired LV function [14]. Aging is associated with decreased LV density and increased EndoMT in LECs, which contribute to tissue fibrosis and functional impairment [40,43]. Given these similarities, we aimed to investigate whether chronic metabolic stress in the db/db mouse model of MetS could also induce EndoMT in cardiac LECs. However, mRNA levels of Snail1 and Snail2—transcription factors responsible for EndoMT—as well as Acta2, which encodes alpha-smooth muscle actin (α-SMA), a marker of mesenchymal transition, were not elevated compared to those from control animals. There was also no decrease in claudin 5 mRNA expression, and immunohistochemical assessment did not reveal the presence of alpha-SMA-positive LECs in myocardial LVs (Figure 4).

We demonstrated that cardiac LECs in db/db mice had increased expression of CPT1A1a, a rate-limiting enzyme in mitochondrial fatty acid β-oxidation [33]. Although the relationship between FAO and EndoMT in LEC has not been studied so far, it can be assumed that, similarly to vascular endothelial cells [44] or cancer cells, increased CPT1A levels modulate the expression of genes associated with EndoMT or EMT, respectively, at the transcriptional and protein levels [45]. In contrast, inhibition of CPT1A activity leads to metabolic reprogramming toward glycolysis, increased oxidative stress, and activation of TGF-β/Smad signaling, which promotes Snail-mediated transcriptional programs associated with EndoMT [40].

Thus, the upregulation of CPT1A in cardiac LECs from db/db mice may reflect a compensatory metabolic adaptation that preserves endothelial identity and prevents the acquisition of mesenchymal characteristics despite chronic metabolic stress. This metabolic resilience could explain the absence of α-SMA expression and the lack of transcriptional activation of Snail1 and Snail2 in these cells.

### 4.4. LV Rarefaction, Oxidative Stress, and Apoptosis

Myocardial hypoxia has been demonstrated in db/db mouse hearts and is considered a contributing factor to cardiac dysfunction [46]. In parallel, the number of LVs is also known to be reduced in these hearts [11]. However, carcinoembryonic antigen-related cell adhesion molecule 1 (CEACAM1) expression in LECs remained unchanged in our model. This observation contrasts with in vitro studies showing that hypoxia inhibits LEC proliferation by downregulating CEACAM1 expression via activation of the JNK pathway and that CEACAM1 silencing mimics the antiproliferative effects of hypoxia [47]. The preserved CEACAM1 expression in our model suggests that the degree of localization of cardiac hypoxia may be insufficient to suppress CEACAM1 in LECs, or that cardiac LECs may exhibit tissue-specific resistance to hypoxia-induced CEACAM1 downregulation.

Oxidative stress arises from an imbalance between prooxidative and antioxidant factors, favoring the former and leading to cellular damage [48]. It is a key contributor to cardiac dysfunction in obesity and MetS, where excess adiposity and insulin resistance promote mitochondrial reactive oxygen species (ROS) production, endothelial injury, and myocardial remodeling [49]. Although oxidative stress is a well-established trigger of apoptosis in endothelial cells, our data indicate that in the myocardium of db/db mice, the expression levels of B-cell CLL/lymphoma 2 (Bcl2) and BCL2 Associated X (Bax) mRNA remain unchanged compared to controls, suggesting that apoptotic cell death is not active at the studied time point. Nevertheless, oxidative stress may still impair LEC function through sublethal mechanisms, such as mitochondrial dysfunction, DNA damage, or impaired proliferation. Recent studies have shown that oxidative stress can sensitize LECs to apoptosis via VEGF-C-dependent pathways, involving increased ROS production, mitochondrial membrane depolarization, and activation of p53 signaling [50]. In our model, the absence of apoptotic gene activation suggests that lymphatic rarefaction may result from non-apoptotic mechanisms, including metabolic exhaustion, mechanical stress due to ECM stiffening, or impaired lymphangiogenic signaling.

In this context, it is noteworthy that the expression of the transcription factor Forkhead Box O1 (FOXO1), a known regulator of LEC proliferation and differentiation, also remained unchanged in our model. FOXO1 has been shown to suppress lymphatic endothelial proliferation and inhibit the expression of valve-forming and differentiation-associated genes, such as PROX1, Forkhead box protein C2 (Foxc2), and GATA2 [51,52]. Its stable expression in cardiac LECs may indicate that FOXO1-mediated transcriptional repression is preserved in the diabetic heart. This could contribute to the reduced number of LV by limiting LEC proliferation and plasticity, thereby impairing compensatory lymphangiogenic responses under metabolic stress.

In our model, we observed increased expression of RELN and emilin-1 in cardiac LECs from db/db mice, while Fbn1 expression remained unchanged. Recent work by Serafin et al. demonstrated that RELN is a lymphangiocrine glycoprotein secreted by LECs in a VE-cadherin-dependent manner and that its secretion is enhanced by adrenomedullin-induced junctional remodeling [53]. Although VE-cadherin expression remained stable in our model, the increased Reln expression may reflect an adaptive response to metabolic stress or altered junctional dynamics in cardiac LECs. The unchanged expression of Fbn1, a structural ECM component, suggests that ECM remodeling in the diabetic heart may be selective, involving regulatory proteins such as emilin-1 and RELN rather than core structural elements. These findings support the hypothesis that lymphatic rarefaction in the diabetic heart may be driven by subtle changes in LEC phenotype and secretory function, rather than overt disruption of junctional integrity or apoptosis.

### 4.5. Immune Cell Trafficking and LEC Dysregulation in MetS Heart

Lymphatic vessels are essential conduits for immune cell trafficking, enabling the transport of dendritic cells, T lymphocytes, and macrophages from peripheral tissues to draining lymph nodes. In the heart, this process is particularly relevant in the context of inflammation and tissue remodeling, where precise regulation of immune cell entry into LVs is critical for maintaining homeostasis [54,55]. Given that the MetS is characterized by chronic, low-grade inflammation and endothelial dysfunction, we assessed mRNA expression for several factors involved in regulating inflammatory cell trafficking through LVs. We observed increased expression of KLF2 and chemokine (C-C motif) ligand 21 (CCL21) mRNA, whereas mRNA levels of endothelial nitric oxide synthase (eNOS), intercellular adhesion molecule 1 (ICAM-1), and chemokine (C-X3-C motif) ligand 1 (CX3CL1) remained unchanged compared with the control group. Importantly, vascular cell adhesion molecule 1 (VCAM-1) mRNA expression was elevated. Adhesion molecules such as VCAM-1 and ICAM-1, expressed on LEC, facilitate the firm adhesion and transmigration of leukocytes across the lymphatic endothelium [56]. Concurrently, chemokines like CCL21 and CX3CL1 create directional cues that guide immune cells toward LVs and influence their retention and activation states [57]. For instance, CX3CL1 modulates the expression and survival of CX3CR1^+^ CD8^+^ T cells, which are involved in tissue surveillance and inflammation resolution [58].

Furthermore, C-C motif chemokine ligand 2 (CCL2) is known to promote lymphangiogenesis by recruiting CCR2^+^ macrophages that secrete lymphangiogenic growth factors [59]. However, in the hearts of db/db mice, the number of LVs is lower than in wild-type mice, and the number of cardiac macrophages is known to be significantly reduced [11,60]. This suggests that despite the presence of chemotactic signals such as CCL21, macrophage recruitment may be impaired in the heart in MetS. Although we did not evaluate this, one possible explanation is the uptake of CCL2 by atypical chemokine receptors such as atypical chemokine receptor 2 (ACKR2), which is expressed by LEC and internalizes inflammatory chemokines, including CCL2 [61,62]. Moreover, PDPN, also elevated in our study, is involved in the presentation of CCL21 on the endothelial surface, facilitating dendritic cell adhesion and transmigration [63].

KLF2 is a key transcriptional regulator that promotes anti-inflammatory endothelial function by upregulating eNOS and suppressing adhesion molecules such as VCAM-1 and ICAM-1 [64,65]. These findings were obtained in primary human umbilical vein endothelial cells (HUVECs), but similar regulatory mechanisms may apply to LEC, given their shared endothelial lineage and responsiveness to inflammatory stimuli. In MetS, however, signaling downstream of KLF2 may be impaired due to altered ERK5 or MEF2 activity (which are required for full transcriptional activation), oxidative stress, or epigenetic modifications, resulting in uncoupling between KLF2 and its canonical targets [64]. The lack of eNOS induction despite elevated KLF2 suggests such a disruption, potentially limiting nitric oxide (NO) production and its anti-inflammatory effects.

Interestingly, although KLF2 is known to suppress VCAM-1 and ICAM-1 expression under physiological conditions, we observed increased VCAM-1 mRNA expression, consistent with a proinflammatory endothelial phenotype commonly observed in cardiovascular disease [65,66,67]. In contrast, the expression levels of ICAM-1 and CX3CL1, which are typically elevated during inflammation, remained unchanged compared with controls. This may reflect tissue-specific regulatory mechanisms or compensatory modulation of immune cell recruitment in cardiac LECs. The increased mRNA expression of CCL21, a chemokine that directs lymphocyte trafficking, may indicate lymphatic remodeling or impaired immune surveillance in the heart affected by MetS.

## 5. Conclusions, Limitations of the Study and Future Research Directions

This study provides novel insights into the transcriptional alterations of cardiac LECs under MetS conditions, using the db/db mouse model. We demonstrate that MetS induces significant changes in mRNA expression of genes involved in lymphangiogenesis, mechanotransduction, immune cell trafficking, and fatty acid metabolism, suggesting a dynamic response of LECs to metabolic and inflammatory stress. However, several limitations should be acknowledged. First, the study focused exclusively on mRNA expression without validating protein levels or assessing functional consequences of the observed transcriptional changes. The exploratory nature of the transcriptomic analysis did not include pathway-specific investigations, which limits mechanistic interpretation. Additionally, the study was conducted at a single time point, which may not capture dynamic changes in LEC phenotype during disease progression. Future studies should incorporate protein-level validation of the key targets identified here to determine whether the observed transcriptional shifts lead to functional changes in cardiac LECs. Based on our dataset, proteins such as CPT1A, GATA2, KLF2, PDPN, VCAM1, SDC4, and EMILIN-1 appear particularly relevant, as they are central regulators of metabolic activity, mechanosensitive signaling, immune cell interactions, and ECM–LEC dynamics. Confirming their expression at the protein level will be crucial to establishing the functional significance of the transcriptional alterations detected in this study. In addition, expanding the experimental design to incorporate multiple time points—particularly early stages before overt symptoms and later stages when metabolic syndrome is fully established—will allow characterization of the temporal dynamics of lymphatic endothelial remodeling. Such analysis could reveal whether observed gene expression changes represent transient adaptive responses or sustained pathological alterations. Furthermore, pathway-focused investigations are needed to dissect signaling mechanisms underlying lymphangiogenesis, mechanotransduction, immune cell trafficking, and lipid metabolism in the cardiac lymphatic endothelium. Given that metabolic syndrome is associated with lymphatic vessel rarefaction, future studies should also aim to clarify the basis of this phenomenon. It remains unknown whether rarefaction results from impaired lymphatic endothelial proliferation, increased apoptosis, or both. Our preliminary findings underscore the importance of lymphatic endothelial responses in cardiac pathology and highlight potential molecular targets for therapeutic intervention in HF associated with metabolic disorders.

## Figures and Tables

**Table 1:** mRNA levels of selected molecules associated with LEC metabolism and survival.

Cellular Pathways	Increased mRNA Expression in db/db Mice	No Differences in mRNA Expressions Between db/db and Control Mice
Mechanosensation and mechanotransduction, matrix stiffness	GATA2, KLF2, PTEN, PDPN, Emillin1, Sdc-4	VE-cadherin, claudin-5, MMP2
EndoMT	CPT1A	Snail1, Snail2, Acta2, claudin 5
Fatty acid β-oxidation	CPT1A	CD36
Lymphatic vessel rarefaction, oxidative stress, and apoptosis	Reln, emilin1	CEACAM1, Bcl2, Bax, FOXO1, Fbn1, VE-cadherin
Immune cell trafficking	KLF2, CCL21, VCAM-1, PDPN	NOS3, ICAM-1, CX3CL1

Abbreviations: Acta2- Actin 2/smooth muscle actin; Bax—BCL2 Associated X; Bcl2—B-cell CLL/lymphoma 2; CCL21—Chemokine (C-C motif) ligand 21; CEACAM1—Carcinoembryonic antigen-related cell adhesion molecule 1; CPT1A—Carnitine Palmitoyltransferase 1A; CX3CL1—chemokine (C-X3-C motif) ligand 1; EndoMT—Endothelial-to-mesenchymal transition; eNOS—endothelial nitric oxide synthase; Fbn1—fibrillin-1; FOXO1—Forkhead Box O1; GATA2—GATA-binding factor 2; ICAM-1—intercellular adhesion molecule 1; KLF2—Krüppel-like Factor 2; MMP2—Metalloproteinase 2; PDPN—podoplanin; PTEN—phosphatase and tensin homolog deleted on chromosome ten; Reln—reelin; Sdc-4—syndecan 4; Snail1—Snail family transcriptional repressor-1; Snail2—Snail family transcriptional repressor-2; VCAM-1—vascular cell adhesion molecule 1; VE-cadherin—Vascular Endothelial Cadherin.

## Data Availability

All raw data generated and analyzed during this study are available from the corresponding author upon request. While the data are not publicly deposited due to institutional policies and data volume constraints, they can be shared with researchers for the purpose of academic collaboration or verification of results. Requests for data should be directed to Justyna Niderla-Bielińska (jniderla@wum.edu.pl).

## Abbreviations

The following abbreviations are used in this manuscript:

ACKR2	atypical chemokine receptor 2
ACTA2	Actin 2/smooth muscle actin
Bax	BCL2 Associated X
Bcl2	B-cell CLL/lymphoma 2
BEC	Blood vascular Endothelial Cells
CCL2	C-C motif chemokine ligand 2
CCL21	Chemokine (C-C motif) ligand 21
CEACAM1	Carcinoembryonic antigen-related cell adhesion molecule 1
CPT1A	Carnitine Palmitoyltransferase 1A
CX3CL1	chemokine (C-X3-C motif) ligand 1
ECM	Extracellular Matrix
EndoMT	Endothelial-to-mesenchymal transition
eNOS	endothelial nitric oxide synthase
FAO	Fatty Acid β-Oxidation
Fbn1	fibrillin-1
FGF2	Fibroblast growth factor 2
Foxc2	Forkhead box protein C2
FOXO1	Forkhead Box O1
FSS	fluid shear stress
GATA2	GATA-binding factor 2
HF	Heart Failure
HFpEF	Heart Failure with Preserved Ejection Fraction
HUVECs	human umbilical vein endothelial cells
ICAM-1	intercellular adhesion molecule 1
KLF2	Krüppel-like Factor 2
LEC	Lymphatic Endothelial Cells
LV	Lymphatic Vessels
LYVE1	Lymphatic vessel endothelial hyaluronan receptor 1
MetS	Metabolic Syndrome
MI	myocardial infarction
MMP2	Metalloproteinase 2
mTORC2	Mechanistic target of rapamycin complex 2
PDGFRβ	Platelet-derived growth factor receptor beta
PDPN	Podoplanin
PI3K	phosphatidylinositol 3-kinase
PROX1	Prospero Homeobox 1
PTEN	phosphatase and tensin homolog deleted on chromosome ten
Reln	Reelin
ROS	Reactive oxygen species
Sdc4	Syndecan 4
Snail1	Snail family transcriptional repressor-1
Snail2	Snail family transcriptional repressor-2
TGF-β	Transforming growth factor beta
VANGL2	VANGL planar cell polarity protein 2
VCAM-1	vascular cell adhesion molecule 1
VE-cadherin	Vascular endothelial cadherin
VEGF-C	Vascular endothelial growth factor C
VEGFR3	Vascular endothelial growth factor receptor 3
α-SMA	α-smooth muscle actin
